# From Primary to Tertiary Care: Expert Position Statements to Guide Heart Failure with Preserved Ejection Fraction Diagnosis

**DOI:** 10.21315/mjms2023.30.1.5

**Published:** 2023-02-28

**Authors:** Wan Azman Wan Ahmad, Azmee Mohd Ghazi, Abdul Kahar Abdul Ghapar, Tamil Selvan Muthusamy, Houng Bang Liew, Imran Zainal Abidin, Mei Lin Ong, Noel Thomas Ross, Yee Ling Cham, Wing Sze Ho, Mayuresh Fegade, David Soon Ping Chew

**Affiliations:** 1 Division of Cardiology, Department of Medicine, University Malaya Medical Centre, Kuala Lumpur, Malaysia; 2 Cardiology Department, The National Heart Institute of Malaysia, Kuala Lumpur, Malaysia; 3 Cardiology Department, Serdang Hospital, Selangor, Malaysia; 4 Cardiology Department, Cardiac Vascular Sentral Kuala Lumpur, Kuala Lumpur, Malaysia; 5 Cardiology Department, Queen Elizabeth Hospital II, Sabah, Malaysia; 6 Department of Medicine, University Malaya Medical Centre, Kuala Lumpur, Malaysia; 7 Cardiology Department, Gleneagles Hospital Penang, Pulau Pinang, Malaysia; 8 Medical Department, Kuala Lumpur Hospital, Kuala Lumpur, Malaysia; 9 Cardiology Department, Sarawak Heart Centre, Sarawak, Malaysia; 10 Novartis Corporation (Malaysia) Sdn. Bhd., Selangor, Malaysia

**Keywords:** heart failure with preserved ejection fraction, diagnosis, natriuretic peptide, echocardiography, cardiac function

## Abstract

Globally, heart failure with preserved ejection fraction (HFpEF) is quickly becoming the dominant form of heart failure (HF) in ageing populations. However, there are still multiple gaps and challenges in making a firm diagnosis of HFpEF in many low-to-middle income Asian countries. In response to this unmet need, the Malaysian HFpEF Working Group (MY-HPWG) gathered and reviewed evidence surrounding the use of different diagnostic modalities indicated for patients with HFpEF to identify diagnostic tools that could be conveniently accessed across different healthcare settings. As a result, five recommendation statements were proposed and an accompanying algorithm was developed, with the aim of improving the diagnostic rate of HFpEF. The MY-HPWG recommends using more easily accessible and non-invasive tools, such as natriuretic peptide (NP) biomarkers and basic echocardiogram (ECHO), to ensure timely HFpEF diagnosis in the primary and secondary care settings, and prompt referral to a tertiary care centre for more comprehensive assessments in uncertain cases.

## Introduction

Heart failure (HF) is a debilitating syndrome that causes reduced functional capacity and quality of life, disability, recurrent hospitalisation and premature mortality; notably, patients with HF often need increasingly intensive care with the deterioration of their condition (1, 2). It has been estimated that > 38 million people worldwide have HF, and the prevalence of HF in the Asia Pacific region ranged from 0.3% to 6.7% (3–5). The total burden of HF—including the utility of healthcare resources and socioeconomic impact—is significant (6); in 2012, approximately USD108 billion per annum was spent on HF globally and this figure is expected to increase substantially in the coming decades (7).

Heart failure with preserved ejection fraction (HFpEF), a subset of HF, is quickly becoming the dominant form of HF in ageing populations worldwide (8). According to a prospective multinational study of patients with HFpEF who were enrolled into the Asian Sudden Cardiac Death in Heart Failure (ASIAN-HF) registry, Asian patients with HFpEF were younger and leaner (i.e. with more than one-third being less than 65 years old and only one-fifth being obese) compared with their Western counterparts; however, they carried a high comorbidity burden, with 70% of patients presenting with at least two comorbidities such as hypertension, anaemia and chronic kidney disease (CKD), among others (8).

At present, there are still multiple gaps and challenges in the management of patients with HFpEF in many low-to-middle income Asian countries, including Malaysia (5, 9, 10). Most national-level disease management guidelines in these countries have been adapted from American or European international guidelines, focusing mainly on the management of comorbidities and may not address the specific needs of the local healthcare system (5, 6). While it has long been accepted that early intervention and subsequent optimisation of treatment in HF lead to improved patient outcomes, the diagnosis of HFpEF—the first step in the management pathway of the disease—remains challenging in resource-limited settings (1). It is thus important to develop localised screening recommendations that take into consideration available resources in different healthcare settings to improve the efficiency of patient diagnosis (1).

**Figure 1:**
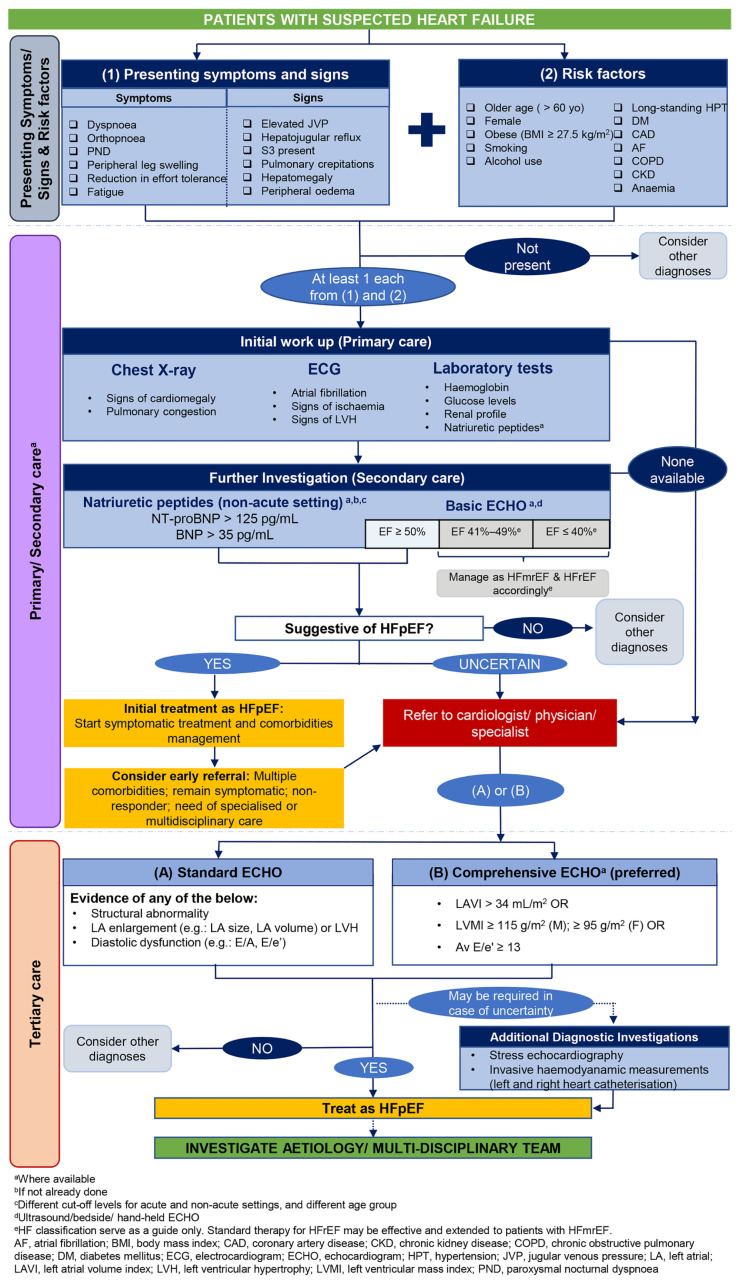
Recommended diagnostic and referral pathway for patients with HFpEF

In response to this unmet need, the Malaysia HFpEF Working Group (MY-HPWG) was convened to collate and review evidence surrounding the use of different diagnostic modalities indicated for patients with HF, with the aim of identifying diagnostic tools that could be conveniently accessed across different healthcare settings. To improve the diagnostic rate of patients at risk of HFpEF who are managed by primary care providers (PCPs), the MY-HPWG proposed five recommendation statements to facilitate the early diagnosis of patients seen at primary and secondary healthcare facilities, and to encourage early referral of patients suspected of HFpEF to tertiary care. An accompanying algorithm was also developed to succinctly present the key message of the recommendations (Figure 1). The recommendations presented in this paper are the opinion of the MY-HPWG based on current clinical evidence and are not meant to replace the use of any official clinical practice guidelines.

## Methods

The MY-HPWG, formed to promote quality management of HFpEF in Malaysia, comprises nine cardiologists and one internal medicine specialist; all of whom have special interest in HF. Additionally, among the cardiologists, two of them have special interest in interventional cardiology, one in heart transplant, one in imaging and one in device therapy. These experts from public, private and university hospitals in Malaysia, were tasked with reviewing current literature on the management of HFpEF (i.e. focusing on the diagnosis of the condition) and synthesising a set of recommendation statements based on collated evidence to guide the diagnosis and referral of patients with HFpEF seen at different healthcare levels in the country.

The MY-HPWG performed a literature review using PubMed (https://pubmed.ncbi.nlm.nih.gov/) and Google Scholar (https://scholar.google.com/) to appraise the value of different diagnostic modalities in diagnosing HFpEF. Relevant studies published between 2004 and 2021 were identified using these search terms: HF, HFpEF, diagnosis, natriuretic peptide (NP), echocardiogram (ECHO) and cardiac function. The search results were then screened for relevance based on the following criteria: i) evidence-based diagnosis of HF/HFpEF; ii) availability of systematic review, meta-analysis or randomised controlled trial and iii) studies of Asian or Malaysian populations.

Subsequently, three expert meetings were convened between September 2020 and June 2021. During the first two meetings, the experts scrutinised the collated data and synthesised unique recommendation statements based on evidence distilled from the literature. A set of five recommendation statements on the diagnosis and referral pathway that apply to patients with HFpEF in Malaysia was proposed. These were discussed and debated extensively during the third expert meeting. The final recommendation statements were unanimously approved and accepted by the MY-HPWG. Meanwhile, an algorithm that reflects the key message of the recommendation statements was also developed concurrently to elucidate the working group’s recommended diagnostic and referral pathway for patients with HFpEF (Figure 1).

### Recommendations

#### Recommendation 1: Recognising Heart Failure in High-Risk Patients

Patients with risk factors presenting with signs and/or symptoms of HF should undergo a detailed medical history review and physical examination to ascertain their risk of HF.

### Signs and symptoms of heart failure

Patients with HF may exhibit nonspecific signs and symptoms (Table 1), thereby complicating its diagnosis (5, 11–13). Notably, many patients with limited exercise capacity and fatigue have normal ejection fraction (EF) on echocardiography (14). On physical examination, features that are more specific for HF include elevated jugular venous pressure (JVP) and the presence of third heart sound (S3) (5, 11, 13).

However, it may be difficult to detect these signs and symptoms in obese individuals (15), older adults (16), and patients with chronic obstructive pulmonary disease (COPD) (17). A prospective analysis showed that patients with HF and higher BMI were less likely to have documented pulmonary crepitations and visible elevation of JVP (15). In older patients (mean age, 82 years) presenting to geriatric outpatient clinics with suspected HF, nocturnal dyspnoea, absence of wheezing, loss of appetite, and lower BMI were independently associated with HF (16). Additionally, both HF and COPD are common in the elderly and share similar signs and symptoms (17). A cross-sectional diagnostic study also revealed that unrecognised HF is common among patients with stable COPD and symptoms of dyspnoea or fatigue with exercise (18).

### Demographics and comorbidities

Patients who are likely to have HFpEF include those with typical demographics (older age and female sex) and comorbid illnesses (Table 2) (19–21). Similarly, a recently published study of patients with HFpEF (*N* = 1,204) enrolled in the ASIAN-HF registry showed that 70% of patients with HFpEF had a least two comorbidities, most commonly hypertension, followed by anaemia, CKD, diabetes mellitus (DM), coronary artery disease (CAD), atrial fibrillation (AF) and obesity (8).

In a Dutch study aimed at screening older patients aged > 60 years old at risk of HF due to type 2 DM (*N* = 581), approximately 32% of patients were found to have previously unknown HF; up to 88% of these patients had HFpEF, while the rest had HF with reduced ejection fraction (HFrEF) (22). Therefore, patients who have at least one element each from Tables 1 and 2 should be actively screened for HF and specifically, HFpEF.

### Identification of patients at risk of HF at all levels of care

Patients with HF often present first in primary care. A population-based study conducted in Canada revealed that more patients with HF were diagnosed in outpatient than in a hospital setting (45.7% versus 36.6%) over a period of 9 years (23), highlighting the critical role that all healthcare providers, at different levels, play in identifying patients at risk of HF. The current Malaysian clinical practice guidelines (CPG) on HF management recommend a shared care model between the hospital (secondary and tertiary) and the community (5). In particular, PCPs play a vital role in identifying individuals with HF via thorough history taking and investigations of HF signs and symptoms (24). Identification and diagnosis of HF in primary care is supported by several international guidelines (11, 13), recommending minimum standards of investigation. Nonetheless, a recent study in the UK demonstrated that limited access to ECHO complicates the diagnosis of HF in the primary care (25). Therefore, PCPs would benefit from a simple and reliable diagnostic algorithm that can be used to facilitate timely diagnosis and intervention.

#### Recommendation 2: Essential Initial Investigations

Patients suspected of having HF should undergo a standard set of assessments, comprising chest X-ray (CXR), electrocardiogram (ECG) and laboratory tests, to establish a provisional diagnosis of HF.

### Utility of standard initial diagnostic work-up

**Figure 2:**
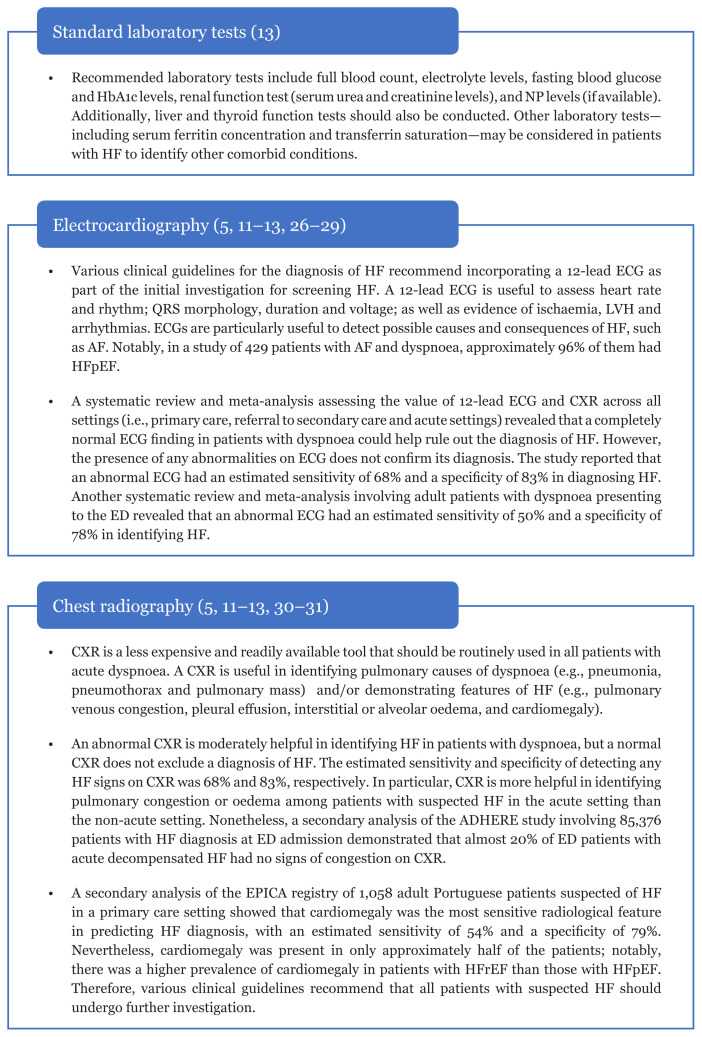
Standard initial diagnostic work-up for routine patient evaluation Notes: ADHERE = Acute Decompensated Heart Failure National Registry; AF = atrial fibrillation; CXR = chest X-ray; ECG = electrocardiogram; ED = emergency department; EPICA = Epidemiology of Heart Failure and Learning; HbA1c = haemoglobin A1c; HFpEF = heart failure with preserved ejection fraction; HFrEF = heart failure with reduced ejection fraction; NP = natriuretic peptide

The scope of initial diagnostic work-up should consider the availability of resources in different healthcare settings (26). Access to echocardiography services in primary and secondary care settings is often limited by the availability of ECHO and appropriately trained personnel. Therefore, simple, non-invasive and less expensive diagnostic procedures, such as standard laboratory tests, CXR and ECG, are more practical for routine patient evaluation in these settings (26). These tests are performed to identify potentially reversible/treatable cause(s) of HF, other aetiologies and comorbidities associated with HF, as well as to evaluate the patient’s suitability for specific therapies (Figure 2) (5, 11–13, 27–31).

#### Recommendation 3: Combining Natriuretic Peptide and Echocardiography to Guide the Diagnosis of Heart Failure

Patients suspected of HF should proceed to additional investigations to confirm their diagnosis with NP measurement and ECHO examination.

### Natriuretic peptides: Brain natriuretic peptide and N-terminal pro-B natriuretic peptide

The use of blood test to measure plasma levels of NPs that is simple and easy to interpret has tremendous clinical significance in many clinical settings (32, 33), especially when HF diagnosis is uncertain and ECHO is unavailable. The determination of plasma NP concentrations enables the selection of patients who should undergo a confirmatory ECHO and allows reasonable exclusion of the initial suspected HF diagnosis in others (34). As such, various clinical guidelines recommend measuring NPs in all patients suspected of HF to facilitate early diagnosis and risk stratification of HF (5, 11–13). Several studies have demonstrated the diagnostic value of NPs (when used alongside a routine history, clinical examination and initial diagnostic investigations) in facilitating the clinical diagnosis of HF (32, 33).

Owing to the high negative predictive value (NPV) but lower positive predictive values in both acute and non-acute settings, the use of NPs is recommended to exclude HF diagnosis, but not to confirm the presence of HF (13). The 2021 consensus statement of Heart Failure Society of America (HFA)/European Society of Cardiology (ESC) and Japanese Heart Failure Society, and 2021 ESC guideline recommended upper limit of normal (ULN) for brain natriuretic peptide (BNP) and N-terminal pro-B natriuretic peptide (NT-proBNP) to rule out HF is summarised in Table 3 (13).

### Interpretation of natriuretic peptide levels

Patients with normal plasma NP concentrations are unlikely to have HF; in contrast, higher plasma NP concentration increases the likelihood that dyspnoea is due to HF (34). Nonetheless, while plasma NP concentrations are usually elevated in most cases of HF, clinicians should be cognisant that elevated plasma NP concentrations have been reported in a range of cardiac and non-cardiac causes (Table 4) (12, 13).

Several studies have shown that NP levels are affected by several factors, such as age (35), obesity (15), AF (36) and renal function (37). NP level increases with increasing age, with or without cardiac comorbidities. A study of 5,508 patients with HF presented at non-acute setting showed that the use of age-stratified NT-proBNP thresholds improved its diagnostic performance considerably over the standard values (35). On the other hand, NP levels are lower in obese individuals with or without HF (15). In order to make an accurate diagnosis in this group of patients, the established cut-off levels of NPs should be lowered by up to 50% (34).

The presence of AF impairs the diagnostic performance of NPs. It is well established that AF is associated with higher concentrations of NPs; these levels may exceed the threshold for ‘HF’ even in the absence of further clinical support for HF diagnosis (34). The analysis of the Biomarkers in Acute Heart Failure (BACH) trial showed that AF was associated with increased plasma NP concentration, even among patients without HF (36). Notably, the cut-off value for NT-proBNP was up to three times higher in patients with AF compared with those without AF in major HFpEF clinical studies like the PARAGON-HF and EMPEROR-PRESERVED trials (38, 39). According to the 2021 ESC guidelines, the ULN in patients with AF are 105 pg/mL for BNP and 365 pg/mL for NT-proBNP, respectively (13).

Similarly, NT-proBNP levels are also affected in patients with renal dysfunction. The estimated glomerular filtration rate (eGFR) is inversely correlated with NT-proBNP level (37); therefore raising the NT-proBNP cut-off value may be necessary to ensure diagnostic accuracy in detecting HF when eGFR is less than 60 mL/min/1.73 m^2^ (34). Nevertheless, given the strong correlation between renal dysfunction and age, no additional adjustment is necessary for NT-proBNP after using age-adjusted rule-in cut-off values (34).

Of note, a cross-sectional analysis of 633 patients with HF revealed that the BNP level was lower for HFpEF than for HFrEF (93 pg/mL versus 266 pg/mL) (40). Compared with the NP cut-off thresholds in existing guidelines, about 18%–29% of patients with HFpEF have normal NP levels (41, 42) and will be missed, highlighting the limitation to the use of BNP in excluding the outpatient diagnosis of HFpEF. Moreover, BNP level is also affected by the direct inhibitory effect of sacubitril/valsartan on neprilysin. Hence, in patients taking sacubitril/valsartan, NT-proBNP is the preferred biomarker to assess the severity of HF and monitor disease progression (34).

### Utility of natriuretic peptide assays

The introduction of point-of-care testing (POCT) devices allows for rapid and robust detection of plasma NP concentration (43). Consequently, this could lead to a quicker investigation of dyspnoea, timely referral and early treatment initiation (44). The diagnostic accuracy of BNP and NT-proBNP POCT in primary and ED settings has been evaluated in a recently published meta-analysis (44). The study reported that NT-proBNP POCT might be more appropriate to exclude HF in the primary care setting, with an estimated sensitivity of 99% and a specificity of 60% at 125 pg/mL (44). In ambulatory care settings, the estimated pooled sensitivity of BNP POCT at 100 pg/mL was 95%, with a specificity of 64%.

### Echocardiography

ECHO is the most useful tool to establish the diagnosis of HF. An ECHO machine can be used to assess at least the left ventricular ejection fraction (LVEF) of patients suspected of HF unless all risk factors (Table 2) are absent or negative (14).

While LVEF measurement may be influenced by several factors (i.e. imaging modality, calculation method and operator factors) (45, 46), it is a commonly used parameter to classify HF and has a consistent predictor of clinical outcomes in HF (45). The 2019 Malaysian CPG on the Management of HF categorised HF into HFrEF, where LVEF ≤ 40%; HFmrEF (defined by the 2021 ESC guideline as HF with mildly-reduced EF), where LVEF 41%–49%; and HFpEF, where LVEF ≥ 50% (5). It is clinically important to distinguish between these diagnoses because their underlying aetiologies, demographics, comorbidities and response to therapies differ (47). Nonetheless, a growing body of literature suggests that standard therapy for HFrEF may be effective in selected patients with HFmrEF (48, 49).

In addition to measuring LVEF, clinical judgement and overall assessment of individual patient is important in the management of HF. A large clinical dataset of patients with LVEF in the USA (*N* = 203,135 patients) showed that deviation of LVEF below 60%–65% was associated with poorer survival irrespective of age, sex or presence of other relevant comorbidities, such as HF; importantly, similar trends were observed for HF patients in both the inpatient and outpatient settings (50). Another large cohort of patients with LVEF in Australia (*N* = 490,155) reported that an LVEF of 60%–64.9% was associated with greater risk of cardiovascular-related mortality in women than men (hazard ratio [HR] = 1.33; 95% confidence interval [CI]: 1.16, 1.52; *P* < 0.001) (51). The American Society of Echocardiography (ASE) and European Association of Cardiovascular Imaging (EACVI) estimate normal LVEF at 62 ± 5% in men and 64 ± 5% in women (52); nonetheless, what constitutes as a ‘normal range’ is also influenced by age and ethnicity (53).

In the ED, focused cardiac ultrasound (FOCUS) has become an indispensable tool to facilitate the diagnostic evaluation of patients suspected of HF. The ASE and American College of Emergency Physicians support the use of FOCUS to primarily assess pericardial effusion, right ventricular enlargement, global cardiac systolic function and volume status (54). A recent study revealed that resident ED physicians could perform FOCUS (on several parameters, such as LVEF, pericardial effusion, right ventricle [RV] pressure overload, regional wall motion abnormalities and RV enlargement) after attending several workshops. The procedure yielded comparable quality to traditional ECHO performed by cardiologists (55). Notably, the high NPV of resident-performed FOCUS can be used as a rule-out test in identifying pericardial effusion, abnormal size and pressure in the RV, cardiac wall motion abnormalities and LVEF (55).

Therefore, FOCUS performed in the ED setting could serve as an objective, rapid and non-invasive tool in assessing patients suspected of HF, especially when cardiologists and ECHO are not readily available.

#### Recommendation 4: Confirming the Diagnosis of Heart Failure with Preserved Ejection Fraction

Patients with uncertain HFpEF diagnosis should be referred to tertiary care for standard/comprehensive echocardiography and/or further investigations.

### Standard echocardiogram

As specified in the 2021 ESC guideline, the criteria for HFpEF diagnosis include the presence of typical signs and symptoms, a preserved EF (LVEF ≥ 50%), elevated levels of BNP (≥ 35 pg/mL) and/or NT-proBNP (≥ 125 pg/mL), as well as objective evidence of cardiac functional and structural alterations which are consistent with HF (56). However, investigative tools to determine such values may not be readily available in the primary or secondary care setting and referral to a higher level of care is often required (26, 57, 58).

At the tertiary care level, a standard ECHO to assess changes in the left ventricular (LV)/left atrial (LA) size or volume and diastolic function is recommended for patients with uncertain HFpEF diagnosis (14). A diagnosis of HFpEF can be confirmed if investigation results meet the following echocardiographic criteria: LVEF ≥ 50% within 72 h of the clinical event; LVH (increased LV wall thickness) or LA enlargement (LA size or volume); and diastolic dysfunction if E/e′ ≥ 13 and the mean e′ septal and lateral wall < 9 cm/s, E/A ≥ 2 or tricuspid regurgitation (TR) velocity > 2.8 m/s (5, 13, 59).

The importance of determining a patient’s diastolic function is highlighted in a retrospective analysis of patients with HFpEF (28). The study demonstrated that the respective univariable sensitivity and specificity of an E/e′ ratio > 9 for HFpEF diagnosis were 78% and 59%, compared with 46% and 86% for E/e′ > 13 (14, 28).

### Comprehensive echocardiogram

In some cases, a standard ECHO may still give inconclusive results and more detailed echocardiographic measurements are required (14). A comprehensive ECHO is recommended owing to its ability to report a patient’s cardiac functional and structural changes more accurately (60, 61).

Primarily, the left atrial volume index (LAVI)—maximal volume of the LA, measured at end-systole from biplane or three-dimensional images, indexed to body surface area (BSA)—should be determined as it is indirectly correlated to LV filling pressure; a marked feature of HFpEF (14). It is also a better marker of chronic LA remodelling than either LA area or diameter (14). A LAVI of > 34 mL/m^2^ is one of the criteria to diagnose HFpEF (5).

**Figure 3:**
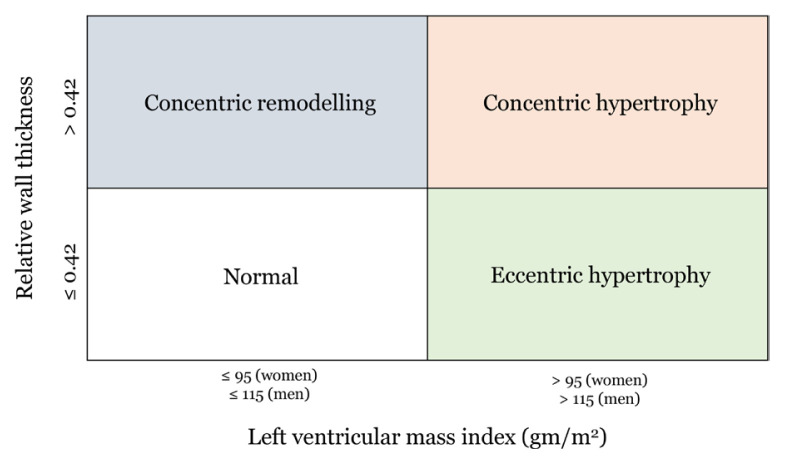
Left ventricle changes categorised according to relative wall thickness and left ventricular mass index. Reprinted by permission of Oxford University Press (52)

Additionally, changes in the left ventricle should be further assessed using relative wall thickness (RWT) and left ventricular mass index (LVMI) normalised to a patient’s BSA or height. According to the Malaysian CPG (5), an LVMI > 115 g/m^2^ for men and > 95 g/m^2^ for women indicate HFpEF, while the ESC categorised LV changes into four different groups (Figure 3) (14, 52). The I-PRESERVE echocardiographic substudy demonstrated the significance of obtaining these values—more than half of the study population (*N* = 745) had either LVH or LV concentric remodelling when LVMI and RWT were measured (62).

### Elevation of filling pressure

Elevation of filling pressure is a hallmark feature of HFpEF and could lead to fluid retention and an expanded plasma volume (14, 63, 64). A recent study found that 58% of the study population who had normal BNP and resting pressure displayed an abnormal increase in left heart filling pressure with exercise, which is consistent with HFpEF (63, 65). In another retrospective analysis, 45% of patients with HFpEF demonstrated elevation in filling pressures only during exercise (early stage HFpEF) (28). These studies revealed an earlier or milder stage of HFpEF that is characterised by normal resting but abnormal exercise haemodynamics, thus suggesting the utility of haemodynamic exercise testing to identify this population of patients (65).

### Stress echocardiogram and invasive haemodynamic measurements

Additional diagnostic investigations such as a stress ECHO may be considered if a diagnosis is still not established (56, 66). The role of stress ECHO in HFpEF diagnosis is supported by a pilot study in which 84.6% of patients suspected with HFpEF developed the condition during exercise. Notably, an E/e′ ratio > 15 during exercise, as detected by diastolic ECHO stress test, has a specificity of 86% in detection of HFpEF, albeit with a low sensitivity (45.5%). Nonetheless, when combined with TR velocity, a value of > 2.8 m/s during exercise provided a significant increase in the sensitivity of HFpEF detection (sensitivity 72.7%, specificity 79.5% and accuracy 78%) (66).

Furthermore, right or left heart catheterisation can also be performed although it may not be universally required for the diagnosis and evaluation of HFpEF. In selected patients, it can be useful in determining cardiac filling pressure at rest and on exercise—an elevated LV filling pressure at rest (LV end-diastolic pressure ≥ 16 mmHg; pulmonary capillary wedge pressure ≥ 15 mmHg) points to a diagnosis of HFpEF (14, 56, 61, 63). However, if these thresholds are not met, assessment of exercise haemodynamics should be conducted (5, 13). This is reflected by the results of a recent study where 45% out of 267 patients had normal filling pressures at rest, but elevated filling pressures were detected during invasive haemodynamic exercise testing (56, 61). In a study to determine the characteristic of right heart dysfunction in HFpEF, right ventricular dysfunction was found to be the strongest predictor of death (HR = 2.4; 95% CI: 1.6, 2.6; *P* < 0.0001) (67).

Information about filling pressure also helps to ascertain the severity of the disease and subsequently, the treatment response. Although cardiac catheterisation remains the gold standard to determine filling pressure owing to its high accuracy that supports early patient diagnosis, it is impractical for all patients presenting with dyspnoea and suspicion of HF to undergo such an invasive procedure (14, 56, 61, 63). Consequently, there is a continuing search for non-invasive markers of elevated LV filling pressure.

### Final aetiology

HFpEF is a heterogeneous condition with various underlying aetiologies and pathophysiological abnormalities—both cardiovascular and non-cardiovascular. Identifying the final aetiology of HFpEF should be part of the diagnostic workup as it allows for the establishment of a possible specific, secondary cause of HFpEF or alternative explanations (13, 56).

Aetiological workup may include a standard exercise stress test that could detect myocardial ischaemia, an abnormal blood pressure response to exercise, chronotropic incompetence, or supraventricular and ventricular arrhythmias. The detection of these conditions could influence management strategies (14). Other more sophisticated tools for aetiological workup are listed in Table 5 (14, 68–73). Identifying the underlying aetiology in HFpEF not only offers specific therapeutic opportunities, but is also associated with the prognosis in HFpEF.

#### Recommendation 5: Timely Referral and Intervention

Patients with HFpEF and multiple comorbidities, non-responders to treatment, or those in need of specialised/multidisciplinary care should be referred to tertiary care for optimal management.

### Reasons for referral

An assessment by a specialist is often recommended to establish a HF diagnosis. In the real world, a large population-based cohort study found that patients with HFpEF were less likely to have a cardiologist as their primary physician and were also less likely to have had a cardiology consultation compared with patients with HFrEF even though both groups had similar rates of in-hospital complications (20).

Most published guidelines, including those from American Heart Association, ESC and National Institute for Health and Care Excellence, state that the diagnosis of HF should be timely and accurate (11–13); adherence to these guidelines is often associated with improved patient outcomes (74). However, a 3-year observational study of clinical practice concluded that only one in five patients with HF was detected in a primary care consultation even though almost half of them presented with indicative symptoms in the year prior to their diagnosis (75). This observation suggests that expert knowledge is essential in making an accurate HF diagnosis (74), thus indicating the need to address the knowledge gap in primary and secondary care settings with a simplified diagnostic algorithm to guide diagnosis and ensure timely specialist referral.

The involvement of a cardiologist to confirm a HFpEF diagnosis is usually necessary owing to the need for a standard or comprehensive ECHO (56, 76). As such, patients with strong suspicion of HFpEF or uncertain HFpEF diagnosis should be referred to tertiary centres for further tests. Importantly, the effective management of HFpEF depends on early diagnosis of the disease (77). Timely detection of diastolic dysfunction and rare diseases – restrictive cardiomyopathies (including cardiac amyloidosis) and constrictive pericarditis – which often lead to HFpEF is essential for an accurate diagnosis (77, 78). Additionally, the latter group of patients may not respond to conventional treatment (79). Timely diagnosis, aetiological work-up and prompt initiation of appropriate therapy allow for treatment optimisation and will subsequently reduce the likelihood of disease progression, hospitalisation and risk of premature death (77, 78).

Patients with HFpEF often have multiple comorbidities which could affect a patient’s prognosis. Thus, the optimal management of comorbidities in HFpEF plays a key role in determining patients’ outcome. A systematic review of 29 randomised trials that investigated strategies to improve patient outcome found that specialised follow-up provided by multidisciplinary healthcare teams significantly reduced mortality rate (relative risks [RR] = 0.75; 95% CI: 0.59, 0.96) in patients with HF compared with regular telephone contact and follow-up by primary care practitioners alone (adjusted RR = 0.82; 95% CI: 0.54, 1.25) (80). Therefore, early referral to tertiary care intervention should be actively considered whenever appropriate/necessary to ensure optimal patient care (76).

## Conclusion

There is an increasing awareness that HFpEF is a heterogenous disease with various phenotypes and comorbidities, making it difficult to establish a diagnosis based on the measurement of LVEF alone. As such, individually tailored approaches to disease diagnosis should be actively considered. The MY-HPWG recommends using more easily accessible and non-invasive tools, such as NP biomarkers and basic ECHO, to ensure timely HFpEF diagnosis in the primary and secondary care settings. Additionally, patients with uncertain diagnosis should be promptly referred to a tertiary care centre to undergo more comprehensive assessments and receive appropriate care.

## Figures and Tables

**Table 1:** Symptoms and signs typical of HF (5, 11–13)

Typical symptoms	More specific signs
Dyspnoea Orthopnoea Paroxysmal nocturnal dyspnoea Peripheral leg swelling Reduction in effort tolerance Fatigue	Elevated jugular venous pressure Hepatojugular reflux Presence of third heart sound Pulmonary crepitations Hepatomegaly Peripheral oedema

**Table 2:** Risk factors and comorbidities associated with HFpEF in symptomatic patients (8, 19–21)

Risk factors	Comorbidities
Older age (aged > 60 years old) Female Obesity (BMI ≥ 27.5 kg/m^2^) Smoking Alcohol use	Long-standing hypertension Diabetes mellitus Coronary artery disease Atrial fibrillation Chronic obstructive pulmonary disease Chronic kidney disease Anaemia

**Table 3:** Definition of HF as per NP measurement (13)

	Acute setting (ULN)	Non-acute setting (ULN)
BNP^a^ (pg/mL)	100	35
NT-proBNP^a^ (pg/mL)	300	125

Notes:

a = Up to 20% of patients with invasively proven HF with preserved ejection fraction have NPs below diagnostic thresholds, particularly in the presence of obesity.

BNP = brain natriuretic peptide; NT-proBNP = N-terminal pro-B natriuretic peptide

**Table 4:** Selected causes of elevated natriuretic peptide concentrations (12, 13)

Cardiac causes	Non-cardiac causes
Heart failure, including right ventricle syndrome Acute coronary syndromes Pulmonary embolism Myocarditis Left ventricular hypertrophy Hypertrophic or restrictive cardiomyopathy Valvular heart disease Congenital heart disease Atrial and ventricular tachyarrhythmias Heart contusion Cardioversion, implantable cardioverter-defibrillator shock Surgical procedures involving the heart	Advanced age Anaemia Ischaemic stroke Subarachnoid haemorrhage Renal failure Liver dysfunction, mainly liver cirrhosis with ascites Pulmonary: Chronic obstructive pulmonary disease, pulmonary hypertension Critical illness Severe burns Severe infections, including pneumonia and sepsis Paraneoplastic syndrome Severe metabolic and hormone abnormalities (e.g. thyrotoxicosis and diabetic ketosis)

**Table 5:** Additional tools for the aetiological workup of cardiac dysfunction (14)

Modality	Function	Examples
Cardiac magnetic resonance (CMR) imaging	Assess cardiac structure and function, stress perfusion imaging, tissue characterisation for fibrosis/oedema	A study to assess the diagnostic and prognostic utility of CMR imaging in 154 patients with HFpEF revealed that 42 (27%) of them had an alternative diagnosis – coronary artery disease, microvascular dysfunction, hypertrophic cardiomyopathy, and constrictive pericarditis. These patients also had worse outcomes (death and HF hospitalisation) after a minimum of 6 months follow-up (median 623 days) (68).
Technetium-99m 3,3-diphosphono-1,2-propanodicarboxylic acid (^99m^Tc-DPD) scintigraphy; technetium-99 m pyrophosphate (^99m^Tc-PYP)	Identify cardiac amyloidosis	The utility of ^99m^Tc-DPD scintigraphy in the diagnosis and subtyping of cardiac amyloidosis has also been investigated in a real-world setting. Remarkably, the selective myocardial uptake of ^99m^Tc-DPD provided a 100% accuracy (95% CI = 97.37, 100) in differentiating transthyretin-related cardiac amyloidosis from light chain cardiac amyloidosis (69). In another cohort of patients with hereditary transthyretin-related cardiac amyloidosis, 99mTc- DPD scintigraphy was able to identify myocardial infiltration across a wide spectrum of morphologic/functional cardiac involvement, resulting in an early diagnosis of the disease (70). In a separate multicentre study, ^99m^Tc-PYP cardiac imaging showed 91% sensitivity and 92% specificity in differentiating patients with transthyretin cardiac amyloidosis from those with amyloid light-chain cardiac amyloidosis and patients with nonamyloid HFpEF (71).
Right or left ventricular myocardial biopsy	Identify various cardiac aetiologies such as cardiac amyloidosis, myocardial fibrosis and myocyte hypertrophy	A large, prospective myocardial tissue analysis of patients with HFpEF found that myocardial fibrosis and myocyte hypertrophy were often present (93% and 88%, respectively) (72). In the same study, 14% of patients were found to have cardiac amyloidosis (72, 73). Tissue analysis in HFpEF could potentially identify key treatable HFpEF phenotypes (72).
Positron emission tomography- computed tomography (PET-CT), specific genetic and laboratory tests	Confirming other specific aetiologies	If CAD is suspected, PET-CT can be conducted. In a study to identify a non-invasive technique for the diagnosis of CAD, results from a PET-CT had 82% positive and 99% negative predictive values of CAD with an accuracy of 97%, suggesting that it has a vital role as a non-invasive procedure to detect patients with CAD for timely therapeutic intervention such as revascularisation (73).

## References

[1] Martinez-Amezcua P, Haque W, Khera R, Kanaya AM, Sattar N, Lam CSP (2020). The upcoming epidemic of heart failure in South Asia. Circ Hear Fail.

[2] Ntiloudi D, Dimopoulos K, Tzifa A, Karvounis H, Giannakoulas G (2021). Hospitalizations in adult patients with congenital heart disease: an emerging challenge. Heart Fail Rev.

[3] Braunwald E (2015). The war against heart failure: the Lancet lecture. Lancet.

[4] Guo Y, Lip GYH, Banerjee A (2013). Heart failure in East Asia. Curr Cardiol Rev.

[5] (2019). Clinical practice guidelines on the management of heart failure.

[6] Rajadurai J, Tse HF, Wang CH, Yang NI, Zhou J, Sim D (2017). Understanding the epidemiology of heart failure to improve management practices: an Asia-Pacific perspective. J Card Fail.

[7] Cook C, Cole G, Asaria P, Jabbour R, Francis DP (2014). The annual global economic burden of heart failure. Int J Cardiol.

[8] Tromp J, Teng TH, Tay WT, Hung CL, Narasimhan C, Shimizu W (2019). Heart failure with preserved ejection fraction in Asia. Eur J Heart Fail.

[9] Lam CSP, Anand I, Zhang S, Shimizu W, Narasimhan C, Park SW (2013). Asian sudden cardiac death in heart failure (ASIAN-HF) registry. Eur J Heart Fail.

[10] MacDonald MR, Tay WT, Teng THK, Anand I, Ling LH, Yap J (2020). Regional variation of mortality in heart failure with reduced and preserved ejection fraction across Asia: outcomes in the ASIAN-HF registry. J Am Heart Assoc.

[11] National Clinical Guideline Centre (UK) (2010). Chronic heart failure: National clinical guideline for diagnosis and management in primary and secondary care [Internet].

[12] Yancy CW, Jessup M, Bozkurt B, Butler J, Casey DE, Drazner MH (2013). 2013 ACCF/AHA guideline for the management of heart failure: a report of the American College of Cardiology Foundation/American Heart Association task force on practice guidelines. J Am Coll Cardiol.

[13] McDonagh TA, Metra M, Adamo M, Gardner RS, Baumbach A, Böhm M (2021). 2021 ESC Guidelines for the diagnosis and treatment of acute and chronic heart failure. Eur Heart J.

[14] Pieske B, Tschöpe C, De Boer RA, Fraser AG, Anker SD, Donal E (2019). How to diagnose heart failure with preserved ejection fraction. The HFA-PEFF diagnostic algorithm: a consensus recommendation from the Heart Failure Association (HFA) of the European Society of Cardiology (ESC). Eur Heart J.

[15] Daniels LB, Clopton P, Bhalla V, Krishnaswamy P, Nowak RM, McCord J (2006). How obesity affects the cut-points for B-type natriuretic peptide in the diagnosis of acute heart failure. Results from the Breathing Not Properly multinational study. Am Heart J.

[16] Oudejans I, Mosterd A, Bloemen JA, Valk MJ, Van Velzen E, Wielders JP (2011). Clinical evaluation of geriatric outpatients with suspected heart failure: value of symptoms, signs, and additional tests. Eur J Heart Fail.

[17] Hawkins NM, Petrie MC, Jhund PS, Chalmers GW, Dunn FG, McMurray JJV (2009). Heart failure and chronic obstructive pulmonary disease: diagnostic pitfalls and epidemiology. Eur J Heart Fail.

[18] Rutten FH, Moons KGM, Cramer MJM, Grobbee DE, Zuithoff NPA, Lammers JWJ (2005). Recognising heart failure in elderly patients with stable chronic obstructive pulmonary disease in primary care: cross sectional diagnostic study. Br Med J.

[19] Andersson C, Vasan RS (2014). Epidemiology of heart failure with preserved ejection fraction. Heart Fail Clin.

[20] Bhatia RS, Tu JV, Lee DS, Austin PC, Fang J, Haouzi A (2006). Outcome of heart failure with preserved ejection fraction in a population-based study. N Engl J Med.

[21] Fonarow GC, Stough WG, Abraham WT, Albert NM, Gheorghiade M, Greenberg BH (2007). Characteristics, treatments, and outcomes of patients with preserved systolic function hospitalized for heart failure. A report from the OPTIMIZE-HF registry. J Am Coll Cardiol.

[22] Boonman-De Winter LJM, Rutten FH, Cramer MJM, Landman MJ, Liem AH, Rutten GEHM (2012). High prevalence of previously unknown heart failure and left ventricular dysfunction in patients with type 2 diabetes. Diabetologia.

[23] Ezekowitz JA, Kaul P, Bakal JA, Quan H, McAlister FA (2011). Trends in heart failure care: has the incident diagnosis of heart failure shifted from the hospital to the emergency department and outpatient clinics?. Eur J Heart Fail.

[24] Gupta M, Bell A, Padarath M, Ngui D, Ezekowitz J (2021). Physician perspectives on the diagnosis and management of heart failure with preserved ejection fraction. CJC Open.

[25] Valk MJ, Mosterd A, Broekhuizen BDL, Zuithoff NPA, Landman MAJ, Hoes AW (2016). Overdiagnosis of heart failure in primary care: a cross-sectional study. Br J Gen Pract.

[26] Mant J, Doust J, Roalfe A, Barton P, Cowie MR, Glasziou P (2009). Systematic review and individual patient data meta-analysis of diagnosis of heart failure, with modelling of implications of different diagnostic strategies in primary care. Health Technol Assess.

[27] Anter E, Jessup M, Callans DJ (2009). Atrial fibrillation and heart failure: treatment considerations for a dual epidemic. Circulation.

[28] Reddy YNV, Obokata M, Gersh BJ, Borlaug BA (2018). High prevalence of occult heart failure with preserved ejection fraction among patients with atrial fibrillation and dyspnea. Circulation.

[29] Wang CS, FitzGerald JM, Schulzer M, Mak E, Ayas NT (2005). Does this dyspneic patient in the emergency department have congestive heart failure?. J Am Med Assoc.

[30] Collins SP, Lindsell CJ, Storrow AB, Abraham WT (2006). Prevalence of negative chest radiography results in the emergency department patient with decompensated heart failure. Ann Emerg Med.

[31] Fonseca C, Mota T, Morais H, Matias F, Costa C, Oliveira AG (2004). The value of the electrocardiogram and chest X-ray for confirming or refuting a suspected diagnosis of heart failure in the community. Eur J Heart Fail.

[32] Booth RA, Hill SA, Don-Wauchope A, Santaguida PL, Oremus M, McKelvie R (2014). Performance of BNP and NT-proBNP for diagnosis of heart failure in primary care patients: a systematic review. Heart Fail Rev.

[33] Roberts E, Ludman AJ, Dworzynski K, Al-Mohammad A, Cowie MR, McMurray JJV (2015). The diagnostic accuracy of the natriuretic peptides in heart failure: systematic review and diagnostic meta-analysis in the acute care setting. BMJ.

[34] Mueller C, McDonald K, de Boer RA, Maisel A, Cleland JGF, Kozhuharov N (2019). Heart Failure Association of the European Society of Cardiology practical guidance on the use of natriuretic peptide concentrations. Eur J Heart Fail.

[35] Hildebrandt P, Collinson PO, Doughty RN, Fuat A, Gaze DC, Gustafsson F (2010). Age-dependent values of N-terminal pro-B-type natriuretic peptide are superior to a single cut-point for ruling out suspected systolic dysfunction in primary care. Eur Heart J.

[36] Richards M, Di Somma S, Mueller C, Nowak R, Peacock WF, Ponikowski P (2013). Atrial fibrillation impairs the diagnostic performance of cardiac natriuretic peptides in dyspneic patients: results from the BACH study (Biomarkers in ACute Heart Failure). JACC Hear Fail.

[37] Takase H, Dohi Y (2014). Kidney function crucially affects B-type natriuretic peptide (BNP), N-terminal proBNP and their relationship. Eur J Clin Invest.

[38] Anker SD, Butler J, Filippatos G, Shahzeb Khan M, Ferreira JP, Bocchi E (2020). Baseline characteristics of patients with heart failure with preserved ejection fraction in the EMPEROR-Preserved trial. Eur J Heart Fail.

[39] Rettl R, Dachs T-MM, Duca F, Binder C, Dusik F, Seirer B (2020). What type of patients did PARAGON-HF select? Insights from a real-world prospective cohort of patients with heart failure and preserved ejection fraction. J Clin Med.

[40] Jorge AL, Rosa MLG, Martins WA, Correia DMS, Fernandes LCM, Costa JA (2016). The prevalence of stages of heart failure in primary care: a population-based study. J Card Fail.

[41] Anjan VY, Loftus TM, Burke MA, Akhter N, Fonarow GC, Gheorghiade M (2012). Prevalence, clinical phenotype, and outcomes associated with normal B-type natriuretic peptide levels in heart failure with preserved ejection fraction. Am J Cardiol.

[42] Obokata M, Kane GC, Reddy YNV, Olson TP, Melenovsky V, Borlaug BA (2017). Role of diastolic stress testing in the evaluation for heart failure with preserved ejection fraction: a simultaneous invasive-echocardiographic study. Circulation.

[43] Clerico A, Prontera C, Emdin M, Passino C, Storti S, Poletti R (2005). Analytical performance and diagnostic accuracy of immunometric assays for the measurement of plasma B-type natriuretic peptide (BNP) and N-terminal proBNP. Clin Chem.

[44] Taylor KS, Verbakel JY, Feakins BG, Price CP, Perera R, Bankhead C (2018). Diagnostic accuracy of point-of-care natriuretic peptide testing for chronic heart failure in ambulatory care: systematic review and meta-analysis. BMJ.

[45] Bozkurt B, Coats AJ, Tsutsui H, Abdelhamid M, Adamopoulos S, Albert N (2021). Universal definition and classification of heart failure: a report of the Heart Failure Society of America, Heart Failure Association of the European Society of Cardiology, Japanese Heart Failure Society and writing committee of the universal definition of heart failure. J Card Fail.

[46] Wood PW, Choy JB, Nanda NC, Becher H (2014). Left ventricular ejection fraction and volumes: it depends on the imaging method. Echocardiography.

[47] Sweitzer NK, Lopatin M, Yancy CW, Mills RM, Stevenson LW (2008). Comparison of clinical features and outcomes of patients hospitalized with heart failure and normal ejection fraction (≥ 55%) versus those with mildly reduced (40% to 55%) and moderately to severely reduced (< 40%) fractions. Am J Cardiol.

[48] Cleland JGF, Bunting KV, Flather MD, Altman DG, Holmes J, Coats AJS (2018). Beta-blockers for heart failure with reduced, mid-range, and preserved ejection fraction: an individual patient-level analysis of double-blind randomized trials. Eur Heart J.

[49] Solomon SD, Vaduganathan M, Claggett BL, Packer M, Zile M, Swedberg K (2020). Sacubitril/valsartan across the spectrum of ejection fraction in heart failure. Circulation.

[50] Wehner GJ, Jing L, Haggerty CM, Suever JD, Leader JB, Hartzel DN (2020). Routinely reported ejection fraction and mortality in clinical practice: where does the nadir of risk lie?. Eur Heart J.

[51] Stewart S, Playford D, Scalia GM, Currie P, Celermajer DS, Prior D (2021). Ejection fraction and mortality: a nationwide register-based cohort study of 499 153 women and men. Eur J Heart Fail.

[52] Lang RM, Badano LP, Victor MA, Afilalo J, Armstrong A, Ernande L (2015). Recommendations for cardiac chamber quantification by echocardiography in adults: an update from the American Society of Echocardiography and the European Association of Cardiovascular Imaging. Eur Heart J Cardiovasc Imaging.

[53] Poppe KK, Doughty RN, Gardin JM, Nagueh SF, Whalley GA, Cameron V (2015). Ethnic-specific normative reference values for echocardiographic LA and LV size, LV mass, and systolic function: the EchoNoRMAL study. JACC Cardiovasc Imaging.

[54] Labovitz AJ, Noble VE, Bierig M, Goldstein SA, Jones R, Kort S (2010). Focused cardiac ultrasound in the emergent setting: a consensus statement of the American Society of Echocardiography and American College of Emergency Physicians. J Am Soc Echocardiogr.

[55] Farsi D, Hajsadeghi S, Hajighanbari MJ, Mofidi M, Hafezimoghadam P, Rezai M (2017). Focused cardiac ultrasound (FOCUS) by emergency medicine residents in patients with suspected cardiovascular diseases. J Ultrasound.

[56] Del Buono MG, Iannaccone G, Scacciavillani R, Carbone S, Camilli M, Niccoli G (2020). Heart failure with preserved ejection fraction diagnosis and treatment: an updated review of the evidence. Prog Cardiovasc Dis.

[57] Raja Shariff RE, Kasim S, Borhan MK, Yusoff MR (2021). Acute heart failure—The ‘real’ Malaysian experience: an observational study from a single non-cardiac centre. Proc Singapore Healthc.

[58] Scott MA, Price CP, Cowie MR, Buxton MJ (2008). Cost-consequences analysis of natriuretic peptide assays to refute symptomatic heart failure in primary care. Br J Cardiol.

[59] Henning RJ (2020). Diagnosis and treatment of heart failure with preserved left ventricular ejection fraction. World J Cardiol.

[60] Gazewood JD, Turner PL (2017). Heart failure with preserved ejection fraction: diagnosis and management. Am Fam Physician.

[61] Ma C, Luo H, Fan L, Liu X, Gao C (2020). Heart failure with preserved ejection fraction: an update on pathophysiology, diagnosis, treatment, and prognosis. Brazilian J Med Biol Res.

[62] Zile MR, Gottdiener JS, Hetzel SJ, McMurray JJ, Komajda M, McKelvie R (2011). Prevalence and significance of alterations in cardiac structure and function in patients with heart failure and a preserved ejection fraction. Circulation.

[63] Borlaug BA, Paulus WJ (2011). Heart failure with preserved ejection fraction: pathophysiology, diagnosis, and treatment. Eur Heart J.

[64] Gori M, Iacovoni A, Senni M (2016). Haemodynamics of heart failure with preserved ejection fraction: a clinical perspective. Card Fail Rev.

[65] Borlaug BA, Nishimura RA, Sorajja P, Lam CS, Redfield MM, Harper AR (2018). Heart failure with preserved ejection fraction. Clin Med J R Coll Physicians.

[66] Belyavskiy E, Morris DA, Url-Michitsch M, Verheyen N, Meinitzer A, Radhakrishnan AK (2019). Diastolic stress test echocardiography in patients with suspected heart failure with preserved ejection fraction: a pilot study. ESC Hear Fail.

[67] Melenovsky V, Hwang SJ, Lin G, Redfield MM, Borlaug BA (2014). Right heart dysfunction in heart failure with preserved ejection fraction. Eur Heart J.

[68] Kanagala P, Cheng ASH, Singh A, McAdam J, Marsh AM, Arnold JR (2018). Diagnostic and prognostic utility of cardiovascular magnetic resonance imaging in heart failure with preserved ejection fraction: implications for clinical trials. J Cardiovasc Magn Reson.

[69] De Haro-Del Moral FJ, Sánchez-Lajusticia A, Gómez-Bueno M, García-Pavía P, Salas-Antón C, Segovia-Cubero J (2012). Role of cardiac scintigraphy with 99mTc-DPD in the differentiation of cardiac amyloidosis subtype. Rev Española Cardiol (English Ed).

[70] Rapezzi C, Quarta CC, Guidalotti PL, Pettinato C, Fanti S, Leone O (2011). Role of 99mTc-DPD scintigraphy in diagnosis and prognosis of hereditary transthyretin-related cardiac amyloidosis. JACC Cardiovasc Imaging.

[71] Castano A, Haq M, Narotsky DL, Goldsmith J, Weinberg RL, Morgenstern R (2016). Multicenter study of planar technetium 99m pyrophosphate cardiac imaging: Predicting survival for patients with ATTR cardiac amyloidosis. JAMA Cardiol.

[72] Hahn VS, Yanek LR, Vaishnav J, Ying W, Vaidya D, Lee YZJ (2020). Endomyocardial biopsy characterization of heart failure with preserved ejection fraction and prevalence of cardiac amyloidosis. JACC Hear Fail.

[73] Namdar M, Hany TF, Koepfli P, Siegrist PT, Burger C, Wyss CA (2005). Integrated PET/CT for the assessment of coronary artery disease: A feasibility study. J Nucl Med.

[74] Ponikowski P, Anker SD, AlHabib KF, Cowie MR, Force TL, Hu S (2014). Heart failure: preventing disease and death worldwide. ESC Hear Fail.

[75] Bottle A, Kim D, Aylin P, Cowie MR, Majeed A, Hayhoe B (2018). Routes to diagnosis of heart failure: observational study using linked data in England. Heart.

[76] Budd JT (2020). Heart failure with preserved ejection fraction: what primary care providers need to know. Consultant.

[77] Naing P, Forrester D, Kangaharan N, Muthumala A, Mon Myint S, Playford D (2019). Heart failure with preserved ejection fraction: a growing global epidemic. Aust J Gen Pract.

[78] Oktay AA, Shah SJ (2015). Diagnosis and management of heart failure with preserved ejection fraction: 10 key lessons. Curr Cardiol Rev.

[79] Pellicori P, Khan MJI, Graham FJ, Cleland JGF (2020). New perspectives and future directions in the treatment of heart failure. Heart Fail Rev.

[80] McAlister FA, Stewart S, Ferrua S, McMurray JJJV (2004). Multidisciplinary strategies for the management of heart failure patients at high risk for admission: a systematic review of randomized trials. J Am Coll Cardiol.

